# Improvement of *In Vitro* Osteogenic Potential through Differentiation of Induced Pluripotent Stem Cells from Human Exfoliated Dental Tissue towards Mesenchymal-Like Stem Cells

**DOI:** 10.1155/2015/249098

**Published:** 2015-01-31

**Authors:** Felipe Augusto Andre Ishiy, Roberto Dalto Fanganiello, Karina Griesi-Oliveira, Angela May Suzuki, Gerson Shigeru Kobayashi, Andressa Gois Morales, Luciane Portas Capelo, Maria Rita Passos-Bueno

**Affiliations:** ^1^Department of Genetics and Evolution, Institute of Bioscience, University of Sao Paulo, 05508-090 Sao Paulo, SP, Brazil; ^2^Science and Technology Institute, Federal University of Sao Paulo, 12247-014 Sao Jose dos Campos, SP, Brazil

## Abstract

Constraints for the application of MSCs for bone reconstruction include restricted self-renewal and limited cell amounts. iPSC technology presents advantages over MSCs, providing homogeneous cellular populations with prolonged self-renewal and higher plasticity. However, it is unknown if the osteogenic potential of iPSCs differs from that of MSCs and if it depends on the iPSCs originating cellular source. Here, we compared the *in vitro* osteogenesis between stem cells from human deciduous teeth (SHED) and MSC-like cells from iPSCs from SHED (iPS-SHED) and from human dermal fibroblasts (iPS-FIB). MSC-like cells from iPS-SHED and iPS-FIB displayed fibroblast-like morphology, downregulation of pluripotency markers and upregulation of mesenchymal markers. Comparative *in vitro* osteogenesis analysis showed higher osteogenic potential in MSC-like cells from iPS-SHED followed by MSC-like cells from iPS-FIB and SHED. CD105 expression, reported to be inversely correlated with osteogenic potential in MSCs, did not display this pattern, considering that SHED presented lower CD105 expression. Higher osteogenic potential of MSC-like cells from iPS-SHED may be due to cellular homogeneity and/or to donor tissue epigenetic memory. Our findings strengthen the rationale for the use of iPSCs in bone bioengineering. Unveiling the molecular basis behind these differences is important for a thorough use of iPSCs in clinical scenarios.

## 1. Introduction

Clinical demand for bone tissue is evident to supplant bony structures lost due to trauma, disease, or congenital malformation. Cell replacement therapies represent a promising strategy for bone engineering, and human mesenchymal stem cells (MSCs) isolated from various adult tissues have been extensively investigated as a potential cell source for bone regenerative treatments [1, 2]. However, large-scale applications are constrained since MSCs are found in limited amounts, are highly heterogeneous, and their long-term in vitro expansion can lead to senescence and spontaneous differentiation [3, 4]. Additionally, the differentiation potential of MSCs may vary depending on the tissue of origin [5].

Generation of human induced pluripotent stem cells (hiPSCs) was first achieved using dermal fibroblasts [6, 7]. Thereafter, hiPSCs have been derived from an ample variety of starting cells, including MSCs. Reprogramming MSCs to hiPSC is an attractive approach to circumvent issues associated with the direct use of MSCs since it allows the production of cells with robust* in vitro* self-renewal capacity and with differentiation multipotential. Controlling differentiation cues* in vivo* is a significant challenge and direct transplantation of pluripotent stem cells may result in tumor formation [8]. Therefore, derivation of MSC-like cells from pluripotent stem cells has been pursued by a number of researchers [9–11].

Most types of MSCs are not easily obtained using minimally invasive procedures. Stem cells from human exfoliated deciduous teeth (SHED) can be easily isolated from a readily accessible tissue source, expanded under simple culture conditions, and banked. Even though SHED have been reported to be especially useful to restore bone [12, 13], as mentioned above, their inherent population heterogeneity and limited expansion capacity restrict their use for therapeutic purposes. While hiPSCs have been generated from SHED (iPS-SHED) [14], there is no report exploring the* in vitro* osteogenic potential of MSC-like cells derived from iPS-SHED populations. Therefore, the goal of this study is threefold: (1) to verify if MSC-like cells from iPS-SHED and SHED isolated from the same donors exhibit similar* in vitro* osteogenic potential; (2) to compare the osteogenic potential of MSC-like cells from iPS-SHED with MSC-like cells from hiPSCs derived from mature dermal fibroblasts (iPS-FIB), considered the most accessible cell source for iPSC generation; (3) to compare the expression of CD105 between these cellular populations, which has been inversely correlated with an increased osteogenic potential [15].

## 2. Materials and Methods

### 2.1. Isolation of Stem Cells from Human Exfoliated Dental Tissue (SHED), Human Dermal Fibroblasts, and Generation of Human Induced Pluripotent Stem Cells (hiPSCs)

SHED were obtained from teeth of 6 independent subjects by enzymatic digestion of pulp from deciduous teeth as described in Miura et al., 2003 [12]. Human adult dermal fibroblasts, the most accessible and feasible cell source for iPSC generation [14], were obtained according to the protocol detailed in Aasen and Belmonte 2010, adapted for fibroblast isolation [16]. hiPSCs were obtained from SHED from 2 independent subjects (3 clones derived from each) and fibroblast cell populations from 3 independent subjects (2 clones each).* SOX2*,* c-MYC*,* OCT4,* and* KLF4* ectopic expression were induced through retroviral transduction, as originally reported in Takahashi et al., 2007 [6]. Two days after transduction, SHED and fibroblasts were cocultivated with irradiated murine embryonic fibroblasts (Millipore) in embryonic stem cell medium Dulbecco's modified Eagle/F12 medium (DMEM/F12) supplemented with 2 mM GlutaMAX-I, 0.1 mM nonessential amino acids, 55 uM 2-mercaptoethanol, 30 ng/mL of bFGF, and 20% of knockout serum replacement all provided by Life Technologies. Typical hiPSC colonies formed on feeder cells were transferred to matrigel (BD-Biosciences) coated plates and expanded in Essential 8 Medium (Life Technologies) supplemented with 100 ug/mL of normocin (Invivogen). hiPSCs displayed embryonic stem cell-like morphology, expressed pluripotency markers (*NANOG, OCT3, OCT4*), and displayed trilineage differentiation potential after embryoid body differentiation and* in vivo* teratoma formation (see Supplementary Figure 1 in Supplementary Material available online at http://dx.doi.org/10.1155/2015/249098). This project was approved by the local ethical committee (Protocol number 121/2001-FR. 435054).

### 2.2. Derivation of MSC-Like Cells from iPS-SHED and iPS-FIB

iPS-SHED and iPS-FIB colonies from confluent plates were detached with accutase (Life Technologies). hiPSC colonies were partially dissociated via manual pipetting and the cells were plated onto matrigel-coated tissue culture dishes at 1 × 10^4^ cells/cm^2^ in MSC differentiation culture medium (Dulbecco's modified Eagle medium High Glucose—DMEM with 10% fetal bovine serum, 1% penicillin/streptomycin, 1% nonessential amino acids, and 5 ng/mL of bFGF) for 14 days with media changes every 3 days. For subsequent passages, single-cell suspensions were prepared using TrypLE reagent (Life Technologies) and cells were passaged with a 1 : 3 split ratio in standard culture flasks (Corning) without matrigel coating.

### 2.3. Characterization of MSC-Like Cells from iPS-SHED and from iPS-FIB

SHED and MSC-like cells from iPS-SHED and from iPS-FIB were harvested and resuspended to 10^5^ cells in 100 uL of PBS containing 1% BSA. Cells were separately labeled with FITC, PE, PE-Cy5, PERCP-Cy5.5, or APC-H7 conjugated rat anti-human antibodies CD29, CD31 (Biolegend), CD34, CD45, CD73, CD90 CD105, and CD166 (Becton Dickinson) on ice and protected from light for 40 min. An isotype-matched mAb was used as a control (Becton Dickinson). Data were acquired and analyzed with the FACSAria II cytometer and CellQuest software (Becton Dickinson). Multipotential differentiations of MSC-like cells from iPS-SHED and from iPS-FIB were performed as previously described by de Mendonça Costa et al., 2008 [13], and representative pictures of adipogenesis, osteogenesis, and chondrogenesis were included as supplementary Figure  2.

### 2.4. Real-Time Quantitative PCR

Total RNA was obtained from cell populations with the use of Nucleospin RNA II extraction kit (Macherey-Nagel) following manufacturer's recommendations. Briefly, one microgram of total RNA was converted into cDNA using Superscript II (Life Technologies), according to the manufacturer's recommendations. Real-time quantitative PCR reactions were performed with 2x SYBR Green PCR Master Mix (Life Technologies) and 25 nM–200 nM of each primer. Fluorescence was detected using ABI Prism 7500 Sequence Detection System, under standard temperature protocol. Primer pairs were designed with Primer-BLAST (http://www.ncbi.nlm.nih.gov/tools/primer-blast/; primer sequences are listed in Table 1, and their amplification efficiencies (*E*) were determined by serial cDNA dilutions log_10_-plotted against Ct values, in which *E* = 10^−1/slope^. Expression of target genes was assessed relative to a calibrator cDNA (ΔCt). Finally, GeNorm v3.4 [17] was used to determine the most stable endogenous controls (among* ACTB, TBP, and HMBS*) and calculate normalization factors for each sample. The final expression values were determined based on a previous method [18], dividing *E* ^−ΔCt^ by the corresponding normalization factor.

### 2.5. *In Vitro* Osteogenic Induction

For osteogenic induction, MSC-like cells from iPS-SHED and from iPS-FIB were plated in 12-well plates (4 × 10^4^ cells per well) and after 3 days, medium was replaced with osteogenic induction medium (Stem Pro Osteogenesis Kit-Life Technologies). Culture medium was changed every 2-3 days and cultures were maintained for 21 days. After 9 days of osteogenic induction, alkaline phosphatase activity was quantified through a biochemical assay: cells were treated with phosphatase substrate (Sigma-Aldrich), and the resulting p-nitrophenol was quantified colorimetrically using a Multiskan EX ELISA plate reader (Thermo Scientific) at 405 nm. After 21 days mineralization of extracellular matrix was assessed through alizarin red staining. Briefly, cells were washed three times with PBS, fixed with a 70% ethanol solution for 30 minutes at room temperature, followed by three distilled water washes, and finally stained with a 0.2% alizarin red S solution (Sigma-Aldrich) for 30 minutes at room temperature. After three washes with PBS, plates were air-dried at room temperature; pictures were taken. Staining was removed with 20% methanol/10% acetic acid solution and measured colorimetrically using a Multiskan EX ELISA plate reader (Thermo Scientific) at 450 nm. von Kossa staining was also performed after 14 and 21 days of osteogenic induction: cell cultures were washed once with PBS, a 1% silver nitrate solution was added, and the plate was exposed to UV light for 40 minutes. After UV light exposure the plate was rinsed with distilled water. Sodium thiosulfate (3%) was added for 5 minutes, the plates were then rinsed in water, and Van Gieson solution was added for 5 minutes. Plates were washed with 100% ethanol and air-dried for image analysis.

### 2.6. Statistical Analysis

All experiments were performed in triplicate. Unpaired Student's *t*-test was used for single comparisons. Error bars in bar graphs represent standard deviation. The level of statistical significance was set at *P* < 0.05.

## 3. Results

**Figure 1:**
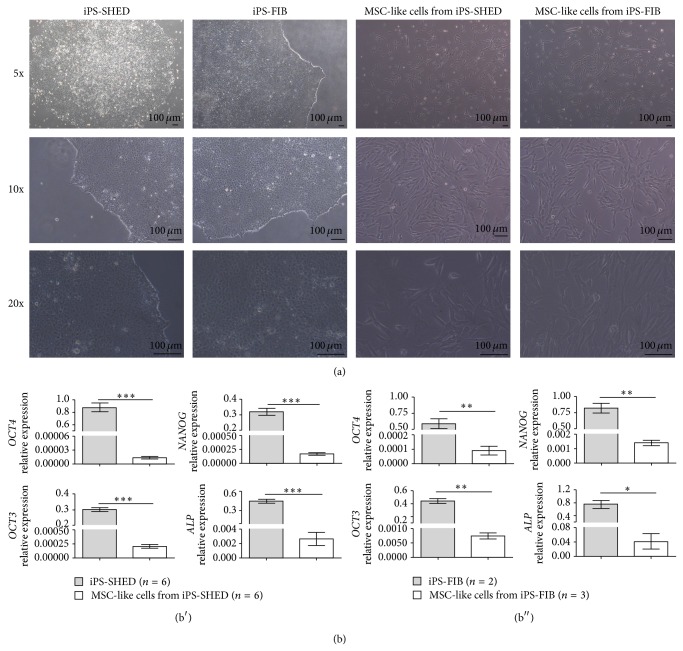
(a) Morphology of undifferentiated hiPSC colonies cultured on matrigel and MSC-like cells from iPS-SHED and iPS-FIB after 12 days of* in vitro* mesenchymal induction. Scale bar = 100 um. (b) Real-time quantitative PCR analysis of pluripotency markers in undifferentiated hiPSCs ((b′) SHED and (b′′) fibroblasts) and in MSC-like cells from iPS-SHED and from iPS-FIB.* ACTB*,* TBP*, and* HMBS* were used as endogenous controls. Values represent means +/− SD, *P* < 0.05 (^*^), *P* < 0.01 (^**^), and *P* < 0.001 (^***^).

**Figure 2:**
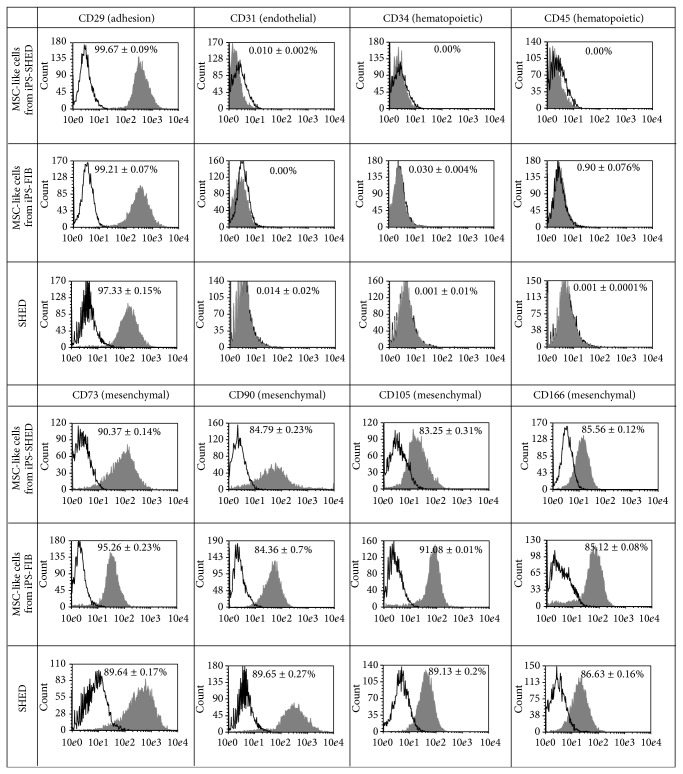
Representative surface antigen profiling of SHED, MSC-like cells from iPS-SHED and from iPS-FIB labeled with antibodies against mesenchymal, endothelial, and hematopoietic antigens. White histograms represent isotype controls and grey histograms represent the fluorescence of conjugated antibodies for each antigen. Mean expression rates are indicated above each graph and displayed as mean +/− SD.

After 12 days of induction of iPS-SHED and iPS-FIB with MSC medium under feeder-free conditions, MSC-like cells derived from iPS-SHED and from iPS-FIB achieved 80% confluence in 25 cm^2^ flasks and showed a spindle-shaped fibroblast-like morphology (Figure 1(a)).* OCT3*,* OCT4*,* NANOG*, and* ALP* mRNAs were significantly downregulated in MSC-like cells from iPS-SHED and from iPS-FIB when compared with the original hiPSC populations (*P* < 0.05, Figures 1(b′) and 1(b′′)). Moreover MSC-like cells from iPS-SHED and from iPS-FIB expressed high levels of mesenchymal markers (CDs 29, 73, 90, and 105 and CD 166) and low levels of endothelial (CD 31) and hematopoietic (CDs 34 and 45) markers (Figure 2).

**Figure 3:**
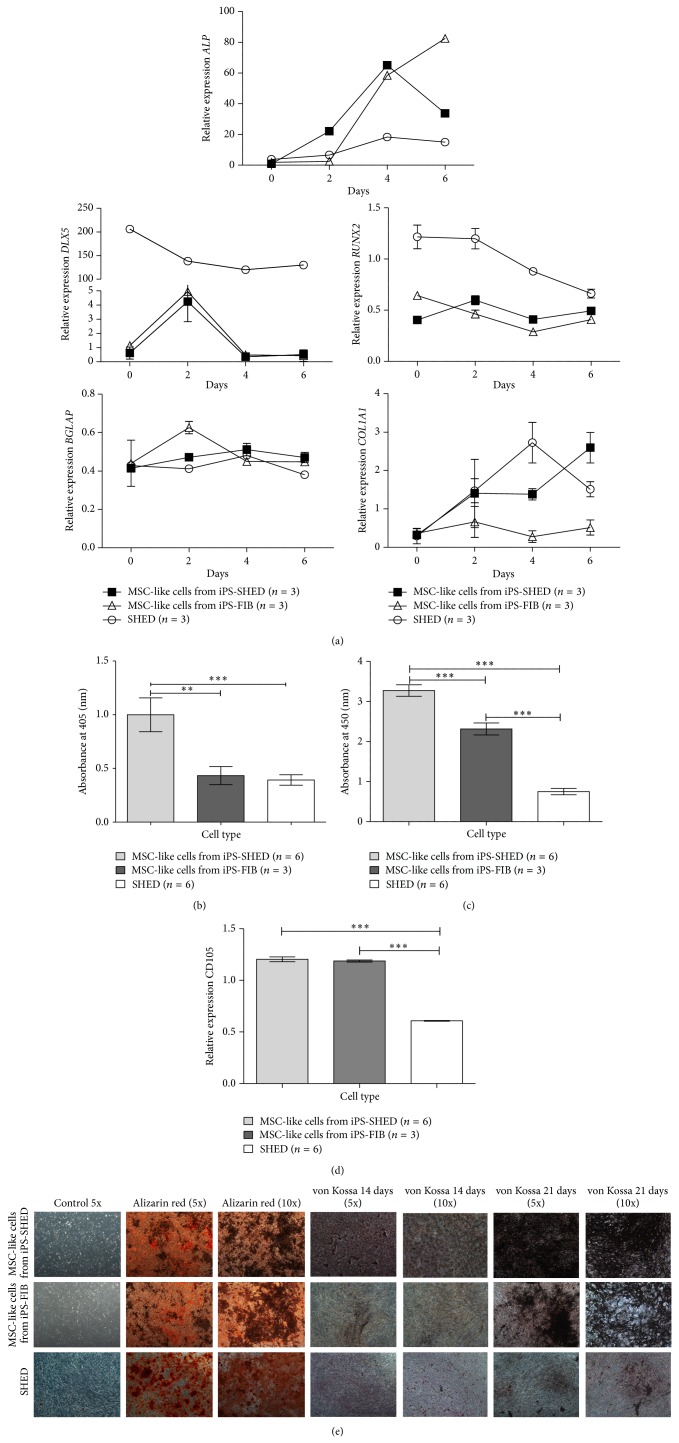
(a) Real-time quantitative PCR analysis of alkaline phosphatase (*ALP*),* DLX5*,* RUNX2*,* BGLAP,* and* COL1A1* in MSC-like cells from iPS-SHED, MSC-like cells from iPS-FIB and SHED.* ACTB*,* TBP*, and* HMBS* were used as endogenous controls. (b) Alkaline phosphatase activity quantification in cells cultured for 9 days in osteogenic medium. Values represent means +/− SD, *P* < 0.01 (^**^), and *P* < 0.001 (^***^). (c) Alizarin red S staining quantification in cells cultured for 21 days in osteogenic medium. Values represent means +/− SD, *P* < 0.001 (^***^). (d) Real-time quantitative PCR analysis of CD105 in undifferentiated SHED and MSCs from iPS-SHED and from iPS-FIB.* ACTB*,* TBP,* and* HMBS* were used as endogenous controls. Values represent means +/− SD, *P* < 0.001 (^***^). (e) Representative pictures of alizarin red S (after 21 days of* in vitro* osteoinduction, with 5 and 10x magnification) and von Kossa staining (after 14 and 21 of* in vitro* osteogenic induction, with 5 and 10x magnification) of mineralized deposits in MSC-like cells from iPS-SHED, MSC-like cells from iPS-FIB and SHED. Basal growth medium free of osteoinduction factors was used in the control group (with 5x magnification).

Next, we assessed the* in vitro* osteogenic potential of MSC-like cells from iPS-SHED, MSC-like cells from iPS-FIB and SHED during early* in vitro* osteogenesis by quantifying gene expression of key osteogenesis markers (*DLX5* and* RUNX2*, two early transcription factors associated with osteogenesis,* ALP* and* COL1A1*, two early osteoblast markers, and* BGLAP*, a late osteoblast marker).* ALP* gene expression was upregulated in all cellular populations from day 2 to day 6 but showed higher expression in days 4 and 6 in MSC-like cells from iPS-SHED and from iPS-FIB in comparison with SHED (*P* < 0.001).* DLX5* peaked at day 2 in MSC-like cells from iPS-SHED and from iPS-FIB and was upregulated in SHED at all time points (*P* < 0.001).* RUNX2* was also upregulated in SHED until day 6 of osteogenic induction, in comparison with MSC-like cells from iPS-SHED and from iPS-FIB (*P* < 0.001).* COL1A1* was upregulated in MSC-like cells from iPS-SHED and in SHED from day 2 to day 6 (*P* < 0.001) but showed no significant upregulation in MSC-like cells from iPS-FIB during this period.* BGLAP* was not upregulated during this early stage of osteoinduction in any cellular population, as expected for a late osteoblast marker (Figure 3(a)).

ALP enzymatic activity was higher in MSC-like cells from iPS-SHED when compared with MSC-like cells from iPS-FIB (2.3-fold increase, *P* < 0.01) and with SHED (2.54-fold increase, *P* < 0.001) after 9 days of* in vitro* osteoinduction (Figure 3(b)). Alizarin red S staining revealed more matrix mineralization in MSC-like cells from iPS-SHED when compared with SHED (4.36- fold increase, *P* < 0.001) and with MSC-like cells from iPS-FIB (1.45-fold increase, *P* < 0.01) after 21 days of osteoinduction (Figure 3(c)). In this time point, MSC-like cells from iPS-FIB showed a 2.99-fold increase (*P* < 0.001) in mineralized matrix production when compared with SHED. These data were validated by von Kossa staining after 14 and 21 days of* in vitro* osteogenesis (Figure 3(e)).

Finally, we compared the expression of* CD105* mRNAs between SHED, MSC-like cells from iPS-SHED and from iPS-FIB and found a lower expression of this gene in SHED when compared with the latter cellular populations (*P* < 0.001, Figure 3(d)).

## 4. Discussion

iPSC technology has gained attention to engender cellular populations to be used in tissue engineering, displaying self-renewal, pluripotency, and differentiation plasticity similar to embryonic stem cells. Furthermore, the use of hiPSCs is not hindered by the ethical issues associated with the use of human embryos and permits the generation of therapeutically relevant cell types genetically compatible to patients, evading rejection drawbacks that may follow transplantation of nonautologous cells [19].

There is an increasing interest in investigating iPSCs for bone regenerative therapies and a series of studies have generated murine iPSCs and assessed their direct differentiation towards osteoblasts [20–23]. From a safety point of view, the use of progenitor cells instead of undifferentiated iPSCs for therapeutic purposes is advantageous since progenitor cells are already primed for a specific differentiation pathway and tumor formation risk is reduced [24]. Moreover, recent reports suggest that some of the reparative effects associated with MSC transplantation are not mediated by cellular differentiation per se but by paracrine factors secreted by them [25]; Fanganiello et al., submitted.

The MSC differentiation from hiPSCs seemed to be successful as both MSC-like cells from iPS-SHED and from iPS-FIB displayed typical mesenchymal cell morphology, downregulation of pluripotency markers and similar cell surface antigen profiles and multipotential when compared with SHED. After* in vitro* osteoinduction, upregulation of osteogenesis markers* DLX5* and* RUNX2* in SHED in comparison with MSC-like cells from iPS-SHED and from iPS-FIB may indicate a previous commitment of this cell population towards the osteogenic lineage. However, in days 4 and 6 of osteoinduction, MSC-like cells from iPS-SHED and from iPS-FIB presented upregulation of* ALP*, a metalloenzyme known as a key early marker of osteogenesis. MSC-like cells from iPS-SHED also had more ALP enzymatic activity when compared with MSC-like cells from iPS-FIB and with SHED in midstage osteogenesis. MSC-like cells from iPS-SHED and from iPS-FIB produced significantly more mineralized extracellular matrix when compared with SHED. Overall, MSC-like cells from iPS-SHED were able to undergo induced* in vitro* osteogenesis in a more efficient fashion than MSCs from iPS-FIB or from the originating SHED populations.

One of the factors that could explain the higher efficiency of the* in vitro* osteogenesis in MSC-like cells from iPS-SHED and iPS-FIB in comparison with SHED might be related to the presence of a more homogeneous cellular population attributed to the direct plating protocol adopted. We have decided to choose the iPSC direct plating method over the embryoid body (EB) protocol since EBs are known to contain a heterogeneous mixture of cells with different degrees of multipotency that may limit their net osteogenic potential [26–28]. Accordingly, enhanced osteogenic differentiation has been associated with direct plating [29–32], and this method has been proposed to yield uniform batches of osteoprogenitor cells [31].

We also tested if the difference in osteogenic potential between the MSC-like cells from iPS-SHED from iPS-FIB and SHED is related to CD105 expression, as its lower expression has been associated with a higher osteogenic potential in MSCs harvested from human adipose tissue (hASCs) when compared with MSCs with higher CD105 expression [15]. Interestingly we found CD105 expression to be significantly lower in SHED when compared with both MSC-like cells from iPS-SHED and from iPS-FIB. Therefore, the higher osteogenic potential in this case may be due to other factors.

The difference in osteogenic potential here reported between MSC-like cells from iPS-SHED and from-iPS-FIB may possibly be related to a somatic epigenetic memory of the tissue of origin [33]. Derivation of pure populations of functionally differentiated cells from iPSCs is still challenging and different cell types show variable susceptibility to reprogramming. In fact, MSCs derived from iPSC lines from different tissues have been shown to exhibit variability in their differentiation profiles. Hynes et al. 2014 reported that MSC-like cells from iPSCs generated from periodontal ligament displayed higher osteogenic capacity both* in vitro* and* in vivo* when compared to MSC-like cells from iPSCs generated from lung and gingival fibroblasts, which was attributed to epigenetic memory of the donor tissue [34]. In another study, Sanchez-Freire et al. 2014 reported higher cardiac differentiation efficiency in MSC-like cells derived from iPSCs generated from cardiac progenitors in comparison with dermal fibroblasts from the same donor, which was demonstrated to be due to the retention of residual methylation signatures of the tissue of origin [35].

## 5. Conclusions

Our findings provide an important argument towards the use of iPSCs in tissue bioengineering since MSC-like cells from iPS-SHED and from iPS-FIB displayed higher osteogenic potential than SHED. We also suggest that cellular homogeneity and tissue of origin are important factors to be considered when planning to use iPSCs in bone regenerative medicine. CD105 does not seem to be a main factor involved in these differences. The dissection of the molecular basis of osteogenic differentiation in MSC-like cells from iPSC-derived cells may furnish insights into the clinical usefulness of iPSCs from different sources.

## Supplementary Material

Supplementary figure 1: Representative images of histopathological sections showing trilineage differentiation potential of human induced pluripotent stem cells stained with hematoxylin and eosin (H&E) after *in vitro* teratoma formation essays in nude mice. Teratomas contained tissues from three germ layers: ectoderm, mesoderm and endoderm.
Supplementary figure 2: Representative pictures of *in vitro* adipogenesis, osteogenesis and chondrogenesis of MSC-like cells from iPS-SHED and from iPS-FIB.

## Figures and Tables

**Table 1:** Primers used for real-time quantitative PCR experiments.

Target	NM	Forward primer	Reverse primer
*OCT3* (pluripotency)	NM_001173531.1	gtggtcagccaactcgtca	ccaaaaaccctggcacaaact
*OCT4* (pluripotency)	NM_002701.3	cctcacttcactgcactgta	caggttttctttccctagct
*NANOG* (pluripotency)	NM_024865	tggacactggctgaatccttc	cgttgattaggctccaaccat
*RUNX2 *	NM_001024630.3	agtggacgaggcaagagtttc	gttcccgaggtccatctactg
*ALP *	NM_000478.4	gatacaagcactcccacttcatctg	ctgttcagctcgtactgcatgtc
*BGLAP *	NM_199173	ggcgctacctgtatcaatgg	gtggtcagccaactcgtca
*COL1A1 *	NM_000088.3	gggccaagacgaagacat	caacacccttgccgttgtcg
*DLX5 *	NM_005221	accagccagaagaagtgac	ccttctctgtaatgcggcca
CD105 (*ENG*)	NM_001144950	tgcacttggcctacaattcca	agctgcccactcaaggatct
*ACTB* (endogenous control)	NM_001101	tgaagtgtgacgtggacatc	ggaggagcaatgatcttgat
*TBP* (endogenous control)	NM_001172085	gtgacccagcatcactgtttc	gcaaaccagaaacccttgcg
*HMBS* (endogenous control)	NM_001024382	agcttgctcgcatacagacg	agctccttggtaaacaggctt
