# Supplementary material for: Improvement of In Vitro Osteogenic Potential through Differentiation of Induced Pluripotent Stem Cells from Human Exfoliated Dental Tissue towards Mesenchymal-Like Stem Cells
Source: Stem Cells Int. 2015 Jan 31;2015:249098. doi: 10.1155/2015/249098 (PMC4329829; doi:10.1155/2015/249098)
Supplement: Supplementary file 1 — Supplementary figure 1: Representative images of histopathological sections showing trilineage differentiation potential of human induced pluripotent stem cells stained with hematoxylin and eosin (H&E) after in vitro teratoma formation essays in nude mice. Teratomas contained tissues from three germ layers: ectoderm, mesoderm and endoderm. Supplementary figure 2: Representative pictures of in vitro adipogenesis, osteogenesis and chondrogenesis of MSC-like cells from iPS-SHED and from iPS-FIB. [file 249098.f1.pdf]

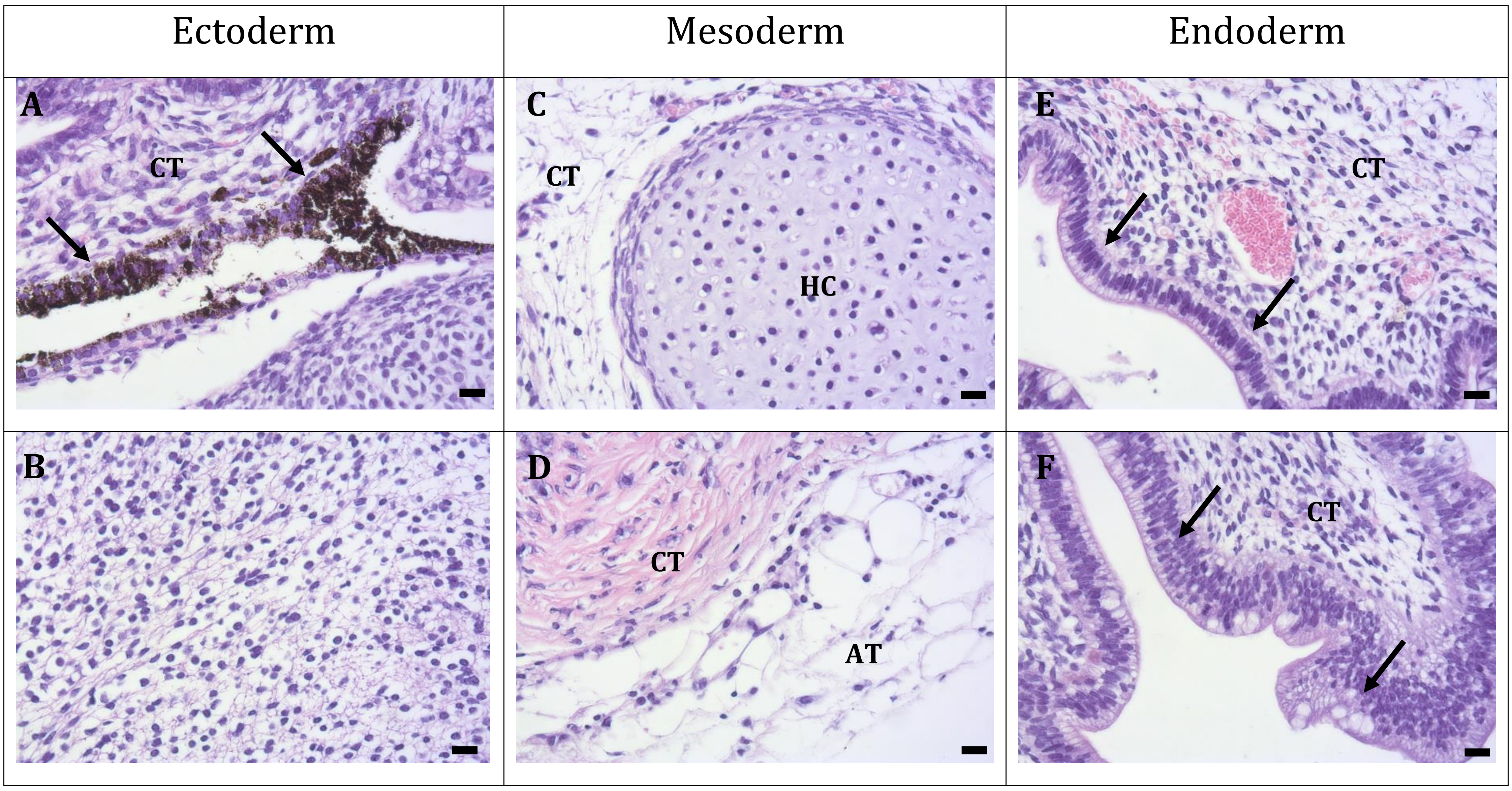

Supplementary figure 1: Hematoxylin and eosin staining of histopathological sections of the iPSC-derived teratoma, containing tissues from the three germ layers. The representative ectodermal tissues include (A) pigment epithelium containing melanin granules and (B) glial cells. The representative mesodermal tissues include (C) hyaline cartilage (HC), connective tissue (CT) and (D) adipose tissue (AT). The representative endodermal tissues include (E) respiratory ciliated epithelium and (F) gastro-intestinal-like epithelium. (A-F) Scale bar= 100ìm (40x magnification). Arrows indicate epithelial tissue.

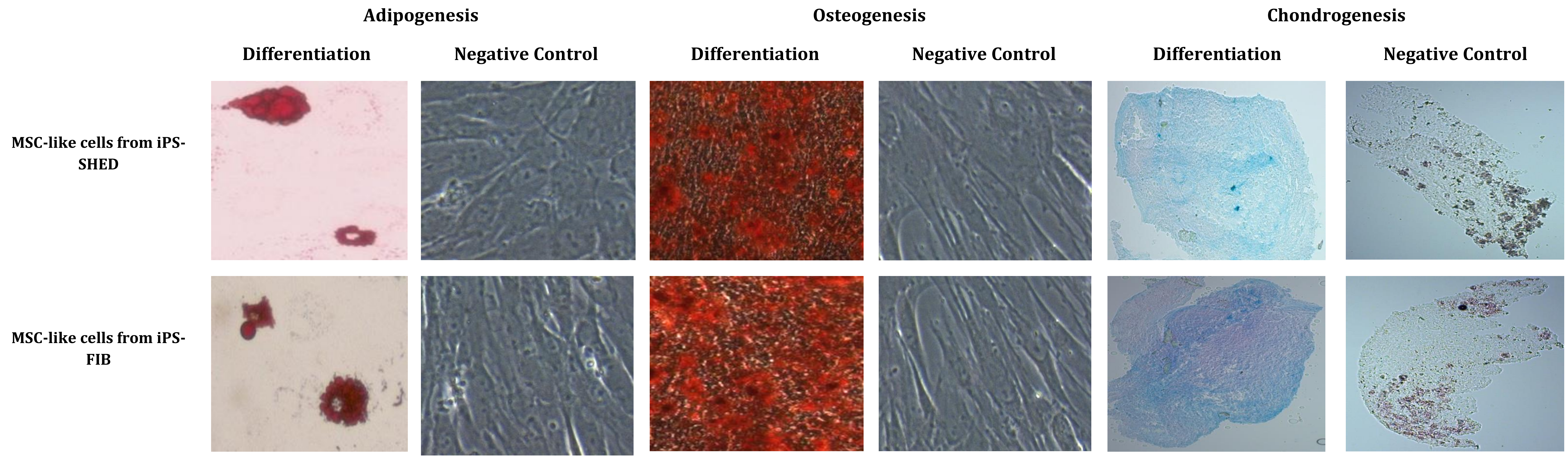

Supplementary figure 2: Representative pictures of adipogenesis (Oil-Red O staining after 14 days of in vitro induction, 20X of magnification), osteogenesis (Alizarin Red S after 21 days of in vitro induction, 20X of magnification) and chondrogenesis (Alcian blue staining after 21 of in vitro induction, 10X of magnification) of MSC-like cells from iPS-SHED and from iPS-FIB.
